# Supplementary material for: Content-rich biological network constructed by mining PubMed abstracts
Source: BMC Bioinformatics. 2004 Oct 8;5:147. doi: 10.1186/1471-2105-5-147 (PMC528731; doi:10.1186/1471-2105-5-147)
Supplement: Additional File 5 — The original Chilibot query results of the term "long-term potentiation (LTP)" and 22 other terms, limiting the latest references analyzed to the years 1990, 1995, 2000, and 2004. [file 1471-2105-5-147-S5.bz2 › chilibotAdditionalFile5/ltp2004/html/PI-3K_PKC.html]

 


 **PI-3K** and **PKC** 
  
Found 314 abstracts in PubMed,  **30 abstracts were retrieved and analyzed**.  


---

 Search Google  |
 PDF files only 
|  EDU domain only 

---

**Interactive relationship** (e.g. stimulation, inhibition, etc)

- Inhibitors of the calcium independent, novel  **PKC**  isoforms... PI3 kinase  [ **PI-3K** ] , or PKA, blocked PMA stimulated cIAP 2 mRNA expression, suggesting a role of  **PKC**  in PMA mediated cIAP 2 induction.  Ref: 14527959 J Biol Chem, 2003
- 2 gliclazide treatment produces a gradual translocation of the diacylglycerol DAG dependent isoforms protein kinase C  **PKC**  alpha, theta, and epsilon from cytosolic to membrane fraction that is dependent on PI3 kinase  [ **PI-3K** ]  and phospholipase C PLC gamma activation.  Ref: 14563700 Endocrinology, 2004
- Functional interactions between cell volume,  **PKC** , and PI 3 kinase  [ **PI-3K** ]  modulate exocytosis thereby regulating ATP release and purinergic signaling in cholangiocytes.  Ref: 14604861 Am J Physiol Gastrointest Liver PhysiolAm J Physiol Gastrointest Liver Physiol, 2004

**Parallel relationship** (e.g. studied together, co-existance, homology, etc.)

- NECA mediated IL 6 release was inhibited by the PLC inhibitor 1 6 17beta 3 methoxyestra, 5 10 tiene 17 yl amino hexyl 1H pyrrole dione, the PI3 kinase  [ **PI-3K** ]  inhibitor wortmannin and the  **PKC**  inhibitors bisindolylmaleimide 1 and bisindolymaleimide X1 HCl Ro 32 0432.  Ref: 14504137 Br J Pharmacol, 2003
- The p38MAPK inhibitor SB203580, the phosphatidylinositol 3 kinase PI3 kinase  [ **PI-3K** ]  pathway inhibitor wortmannin, and the protein kinase C pathway  **PKC**  inhibitor Gö 6976 did not significantly affect mitogen induced MCM6 and MCM7 expression.  Ref: 14516785 Exp Cell Res, 2003
- Furthermore, insulin induced GSK 3beta phosphorylation was mediated by both PI 3 kinase  [ **PI-3K** ]  PKB and the TC10 Par6 atypical  **PKC**  signaling pathways.  Ref: 14734537 J Cell BiolJ Cell Biol, 2003
- The negative modulator effects of  **PKC**  on integrin expression and PI 3 kinase  [ **PI-3K** ]  activation correlated with its ability to limit the HGF induced motogen response.  Ref: 14709339 Cell Signal, 2004
- The ceramide induced augmentation of PS was not significantly affected by inhibition of phosphatidylinositol PI 3 kinase  [ **PI-3K** ] , protein kinase C  **PKC** , ceramide activated protein phosphatase CAPP, and nitric oxide NO synthase.  Ref: 14645138 Br J Pharmacol, 2003
- PI 3 kinase  [ **PI-3K** ] , phospholipase C gamma, and the conventional  **PKC**  beta 2 seem to be involved in DHEA effects.  Ref: 14693696 Diabetes, 2004
- These data suggest that SOM treatment of spleen cells, especially in CD8 T cells, leads to downregulation of LFA 1 mRNA translation, inside out signaling molecules for integrins Ras, Rap1 and PI 3 kinase  [ **PI-3K** ] ... and consequently to a decrease in the LFA 1 mediated adhesion to ICAM 1.  Ref: 14758054 Neuroimmunomodulation, 2004
- The effects of DHEA are abrogated by pretreatment of adipocytes with PI 3 kinase  [ **PI-3K** ]  and phospholipase C gamma inhibitors, as well as by inhibitors of calcium dependent  **PKC**  isoforms, including a specific  **PKC**  beta inhibitor.  Ref: 14693696 Diabetes, 2004
- These data establish that GP Ib IX V itself can signal to activate alphaIIbbeta3, through sequential actions of Src kinases, calcium oscillations, and PI 3 kinase  [ **PI-3K** ]   **PKC** .  Ref: 14726383 BloodBlood, 2004
- Both vesicular exocytosis and ATP release were proportional to cell volume, and both exhibited similar regulatory properties including 1 dependence on intact PI 3 kinase  [ **PI-3K** ] , 2 attenuation by inhibition of protein kinase C  **PKC**  and 3 potentiation by activation of protein kinase C prior to hypotonic exposure.  Ref: 14604861 Am J Physiol Gastrointest Liver PhysiolAm J Physiol Gastrointest Liver Physiol, 2004
- Studies using inhibitors of the EGF receptor tyrosine kinase, phosphatidylinositol 3 kinase PI3 kinase  [ **PI-3K** ]  and protein kinase C  **PKC**  revealed that all of the cell surface agonists could activate PAK1 through pathways independent of  **PKC** , that EGF stimulated a PI3 kinase  [ **PI-3K** ]  dependent pathway to stimulate PAK1,.  Ref: 14575865 Cell Signal, 2003
- However, these insulin induced events are uniquely controlled by PI3 kinase  [ **PI-3K** ]  and  **PKC**  zeta, which act upstream of Aurora A.  Ref: 14724178 Genes Dev, 2004
- PI3 kinase  [ **PI-3K** ]  products could induce PLC gamma activation, whose hydrolytic activity could activate the DAG dependent isoforms  **PKC**  alpha, theta, and epsilon.  Ref: 14563700 Endocrinology, 2004
- METHOD To define the mechanism of the movement of leukocytes to the inflammatory site by somatostatin SOM and substance P SP, we examined the expression of the adhesion molecule LFA 1 and inside out signals for integrins, protein kinase C  **PKC** , Ras, Rap1, and phosphoinositide PI 3 kinase  [ **PI-3K** ] , in anti CD3, anti CD3 SOM, anti CD3 SP stimulated or unstimulated spleen cells.  Ref: 14758054 Neuroimmunomodulation, 2004
- DAF upregulation was mediated via a protein kinase C  **PKC**  alpha, phosphoinositide 3 kinase PI 3 kinase  [ **PI-3K** ] , p38 mitogen activated protein kinase MAPK and nuclear factor kappaB NF kappaB dependent pathway.  Ref: 14511240 Immunology, 2003
- To study signaling pathways mediating MEF2 activity, human skeletal muscle cell cultures or isolated rat epitrochlearis muscles were pre incubated with inhibitors of p38 mitogen activated protein kinase MAPK 10 micro M SB203580, MEK1 50 micro M PD98059, protein kinase C  **PKC**  1 and 10 micro M GF109203X, phosphatidylinositol PI 3 kinase  [ **PI-3K** ]  10 micro M LY294002, or AMP activated protein kinase AMPK 20 micro M Compound C.  Ref: 14960415 Am J Physiol Cell PhysiolAm J Physiol Cell Physiol, 2003
- To the contrary, the decrease of PI 3K  [ **PI-3K** ]  activation, which followed the HGF induced increase was not accompanied by the loss of phosphotyrosine docking sites and was prevented by the inhibition of  **PKC** .  Ref: 14709339 Cell Signal, 2004
- These findings suggest that intracellular tyrosine kinases, MEK ERK and PI3 kinase  [ **PI-3K** ]  AKT pathways are involved in 5 S HETE stimulated pancreatic cancer cell proliferation but P38 kinase, JNK SAPK and  **PKC**  are not involved in this mitogenic effect.  Ref: 14707447 Oncology, 2003
- Assays with inhibitors and antibodies against proteins involved in signal transduction demonstrated that NAPS and TAPS elevated COX 2 expression via tyrosine kinase, src, PI 3 kinase  [ **PI-3K** ]  and  **PKC** , followed by ERK activation.  Ref: 14708615 J Invest Dermatol, 2003
- Our data suggest that ceramide acutely augments the contractile function of cardiac myocytes through an alternative mechanism s rather than PI 3 kinase  [ **PI-3K** ] ,  **PKC** , CAPP, or NO.  Ref: 14645138 Br J Pharmacol, 2003
- In this study, Mouse long bone cells including osteoclasts and osteoblast were treated with the PI3 kinase  [ **PI-3K** ]  inhibitor, wortmannin WT, and a specific inhibitor of protein kinase C  **PKC** , calphostin C.  Ref: 14555293 Int Immunopharmacol, 2003
- Pharmacological inhibition of protein kinase C  **PKC**  or PI 3 kinase  [ **PI-3K** ]  prevented alphaIIbbeta3 activation but not calcium oscillations.  Ref: 14726383 BloodBlood, 2003
- Exposure of cells to osmotic or oxidative stress increased MEF2 DNA binding via pathways that were completely blocked by MAPK inhibitors, and partially blocked by inhibitors of  **PKC** , PI 3 kinase  [ **PI-3K** ] , and AMPK.  Ref: 14960415 Am J Physiol Cell PhysiolAm J Physiol Cell Physiol, 2003
